# Nodal follicular dendritic cell sarcoma with four atypical histomorphologic features: an unusual case report

**DOI:** 10.1186/s13000-019-0869-2

**Published:** 2019-08-14

**Authors:** You-Li Wu, Feng Wu, Robinson Ateh Mbako, Yu Zhang, Guang-Jie Duan, Xiao-Chu Yan

**Affiliations:** 1 Institute of Pathology and Southwest Cancer Center, Southwest Hospital, Third Military Medical University (Army Medical University), Chongqing, 400038 China; 2 Department of Internal Medicine, University Hospital Laquintinie and Faculty of Medicine, Douala, Cameroon

**Keywords:** Follicular dendritic cell sarcoma, Histomorphology, Variants, Diagnosis

## Abstract

**Background:**

Follicular dendritic cell sarcoma (FDCS) is a rare malignancy. In addition to the classical histopathologic features, it has also some special morphological variants that can present a challenge in the diagnosis of this disease.

**Case presentation:**

A 45-year-old male who presented with a left supraclavicular mass was given a final diagnosis of FDCS after lymph node biopsy. The specimen obtained during radical resection revealed five different morphologies, including the classical histological appearance and atypical areas resembling desmoplastic infiltrative carcinoma, anaplastic large cell lymphoma (ALCL), hemangiopericytoma and classical Hodgkin’s lymphoma (CHL). Immunohistochemistry was notable for positive CD21 and CD23 expression across all morphologies. Given the atypical appearance and location, the specimen was initially misdiagnosed as a metastatic carcinoma based on histology alone at an outside institution. The patient eventually underwent surgical resection followed by adjuvant chemotherapy and radiation. Despite treatment, the disease progressed, and the patient passed away 36 months after surgery.

**Conclusions:**

This unusual case of FDCS contains four types of atypical histomorphologies within a single tumor specimen, including those resembling ALCL and hemangiopericytoma which are described here for the first time. Our report further expands the histopathologic spectrum of FDCS and may help assist in the diagnosis of other such challenging cases.

## Background

Follicular dendritic cell sarcoma (FDCS), an uncommon malignancy found primarily in cervical lymph nodes, is characterized by its follicular dendritic cell (FDC) differentiation [1]. The typical morphology of FDCS includes ovoid- to spindle-shaped tumor cells arranged in fascicular, storiform or whorled patterns; these tumor cells have indistinct cellular borders with lightly eosinophilic cytoplasm, delicate nuclear membranes with granular or vesicular chromatin, and small but distinct nucleoli. In the background, small lymphocytes can often be noted throughout the tumor [2, 3]. However, when these typical morphologies are not present, the diagnosis can be more challenging. Previous studies have reported cases of FDCS that resemble poorly differentiated carcinoma [3, 4]. A biopsy can show epithelioid and pleomorphic tumor cells with marked nuclear atypia; when these round or polygonal epithelioid cell nests appear in lymph nodes, the tumor is remarkably similar to metastatic carcinoma, especially in the appropriate clinical context.

Here, we report an unusual case of FDCS in the left supraclavicular lymph node. In addition to the classical histologic features, it also exhibits four atypical morphologies: those mimicking desmoplastic infiltrative carcinoma, anaplastic large cell lymphoma (ALCL), hemangiopericytoma and classical Hodgkin’s lymphoma (CHL). These atypical histological features pose a great challenge to diagnosis. This case extends the morphologic spectrum of FDCS and furthers understanding of this rare tumor.

## Case presentation

A 45-year-old male presented with a hard, fixed, 4 cm mass in the left supraclavicular fossa without pain, redness, swelling, or fever. Ultrasound revealed multiple enlarged lymph nodes that were not palpable by physical exam and that were subsequently biopsied. The initial diagnosis was metastatic poorly differentiated carcinoma, and evaluation for a primary tumor was recommended. The initial evaluation was performed at an outside institution; the patient was not a surgical candidate and was subsequently referred to our center for further treatment.

**Fig. 1:**
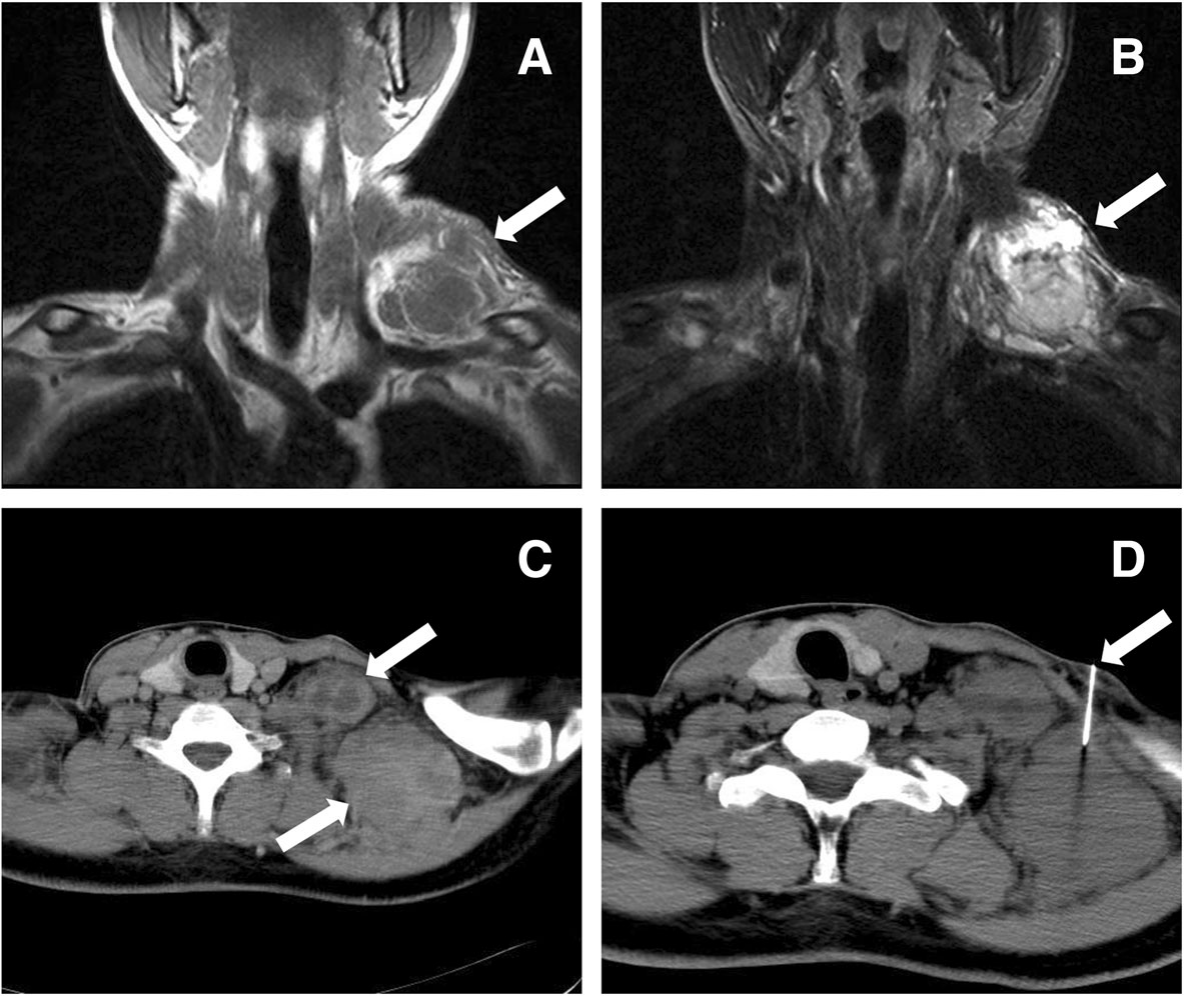
T1-weighted images (**a**) and T2-weighted coronal images (**b**) on MRI show a soft tissue mass with inhomogeneous densities and fine separations in the left supraclavicular fossa (arrows). Axial CT scan (**c**) reveals a fused multinodular mass (arrows) with local necrosis; CT-guided core needle biopsy was then performed (**d**)

After a thorough evaluation, no suspicious primary lesion was found. Routine laboratory tests, including a comprehensive metabolic panel and detection of multiple tumor markers (e.g., lactate dehydrogenase / LDH), were within normal limits. Computed tomography (CT) of the chest, electronic nasopharyngoscopy, gastrointestinal endoscopy and abdominal color Doppler ultrasound were all unremarkable. Magnetic resonance imaging (MRI) revealed a 9.0 cm × 6.5 cm × 5.0 cm soft tissue mass with inhomogeneous densities and fine separations in the left supraclavicular fossa (Fig. 1a, b). Due to this unremarkable workup, a second lymph node biopsy was performed under CT guidance to confirm the diagnosis (Fig. 1c, d).

**Fig. 2:**
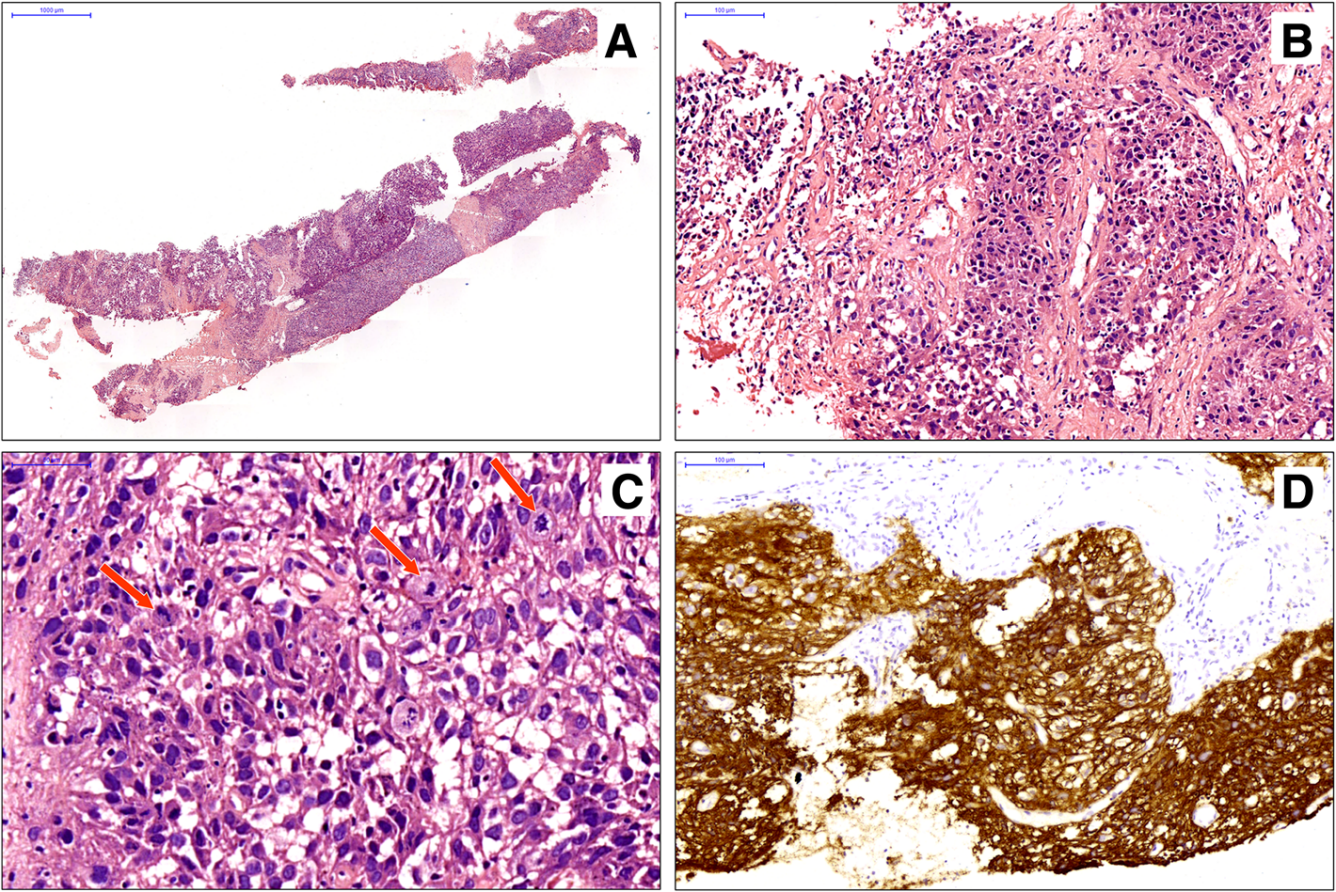
Histopathological and immunohistochemical features of the core needle biopsy specimen: **a** Low-power view of the needle biopsy specimen. **b** Atypical epithelioid tumor cell nests infiltrating the hyperplastic collagen fibers mimic a metastatic carcinoma. **c** These tumor cells are round to polygonal with atypical mitoses (arrows) and are positive for CD23 (**d**)

Histopathological examination showed round to polygonal cells in a nested pattern with atypical mitoses and focal areas containing many small lymphocytes, which suggested metastatic carcinoma (Fig. 2a-c). However, the immunohistochemistry (IHC) results were negative for pan cytokeratin (CK AE1/AE3), CK5/6, CK7, CK20, CK8/18, TTF1, P63, CDX-2, Syn, CgA, LCA, HMB45 and S-100 protein; only vimentin was positive. Thus, the initial diagnosis was doubted. Further staining demonstrated CD21 and CD23 positivity (Fig. 2d), weak positivity for epithelial membrane antigen (EMA), and negative expression of CD35, CD30, CD34, smooth muscle actin (SMA), Desmin, SALL4, CD10, TFE3, and MyoD1. Given this immunophenotype, FDCS was diagnosed. A review of the biopsy specimen from the outside institution confirmed these findings.

**Fig. 3:**
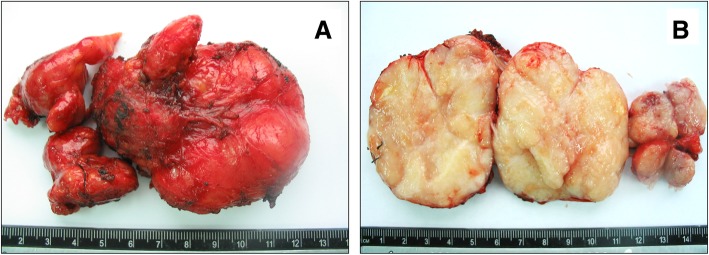
Gross pathology of the resected tumor shows the fused mass is well circumscribed with an intact capsule (**a**) and that the cut surface is yellow-gray and tan with areas of focal hemorrhage (**b**)

A bone scan revealed no abnormalities, and the patient refused further imaging by positron emission tomography and CT (PET-CT). A multidisciplinary conference determined the lesion was most likely confined to the neck with no metastasis. Thus, radical resection of the tumor was performed. The mass was well-circumscribed and appeared to be composed of several nodules of different sizes fused together, the largest of which measured 7 cm × 5 cm × 4 cm and the smallest of which measured approximately 1 cm × 1 cm × 0.5 cm. The cut surface was yellow-gray and tan with areas of focal hemorrhage (Fig. 3a, b).

**Fig. 4:**
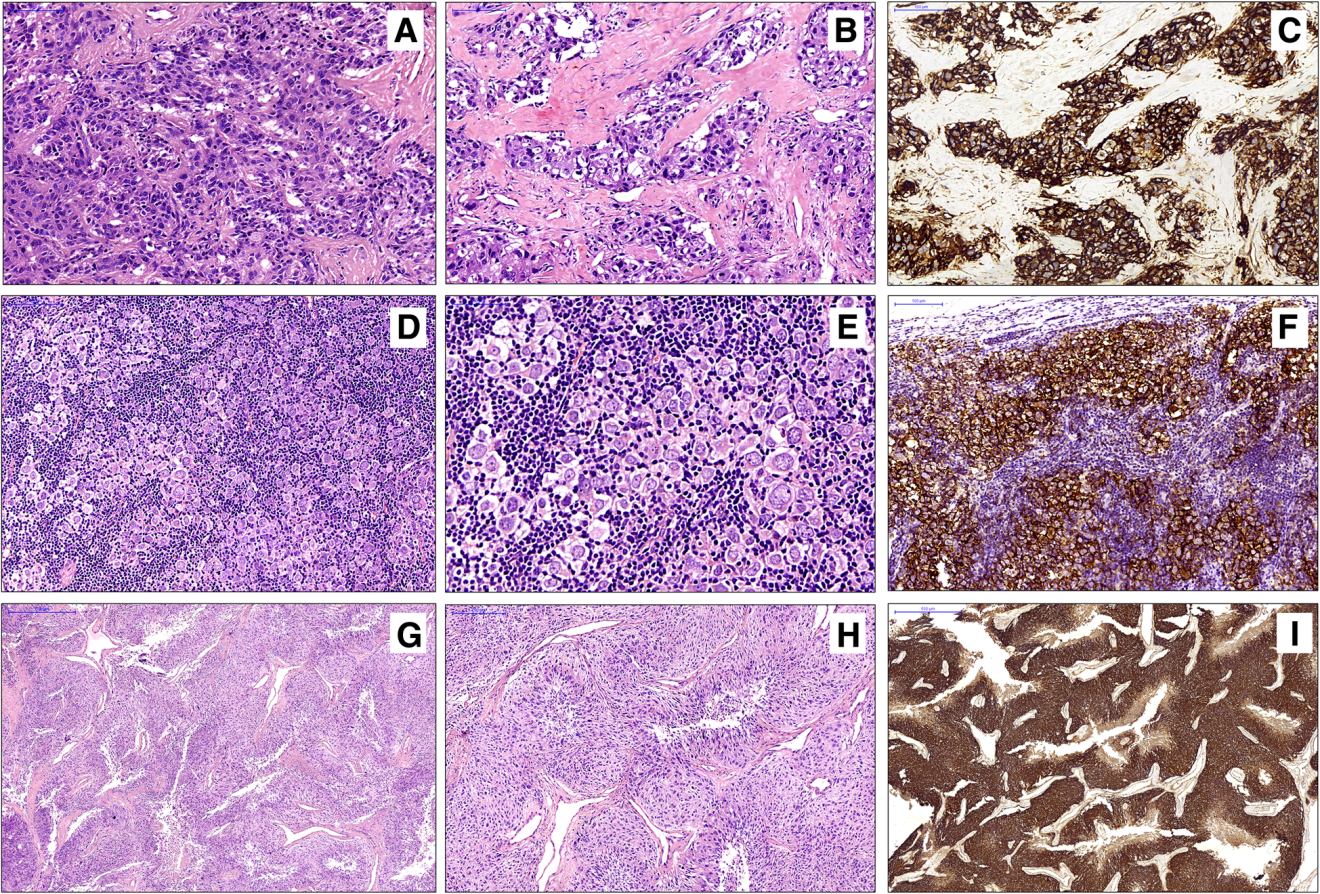
**a**-**c**: The area resembles desmoplastic infiltrative carcinoma. Atypical epithelioid tumor cells arranged in nested or pseudo-glandular structures (**a**) infiltrate the hyperplastic collagen fibers (**b**), which are positive for CD23 (**c**). **d**-**f**: The area resembles anaplastic large cell lymphoma. Atypical large tumor cells with a nested or patchy pattern fill the lymphatic sinuses (**d**). On high power, the tumor cells are round or polygonal with marked atypia (**e**) and are positive for CD21 (**f**). **g**-**i**: The area mimics hemangiopericytoma. Short spindle-shaped tumor cells are arranged radially around many fissure-like or dendritic thin-walled blood vessels (**g**) with only a few infiltrating lymphocytes (**h**), which are positive for CD23 (**i**)

**Fig. 5:**
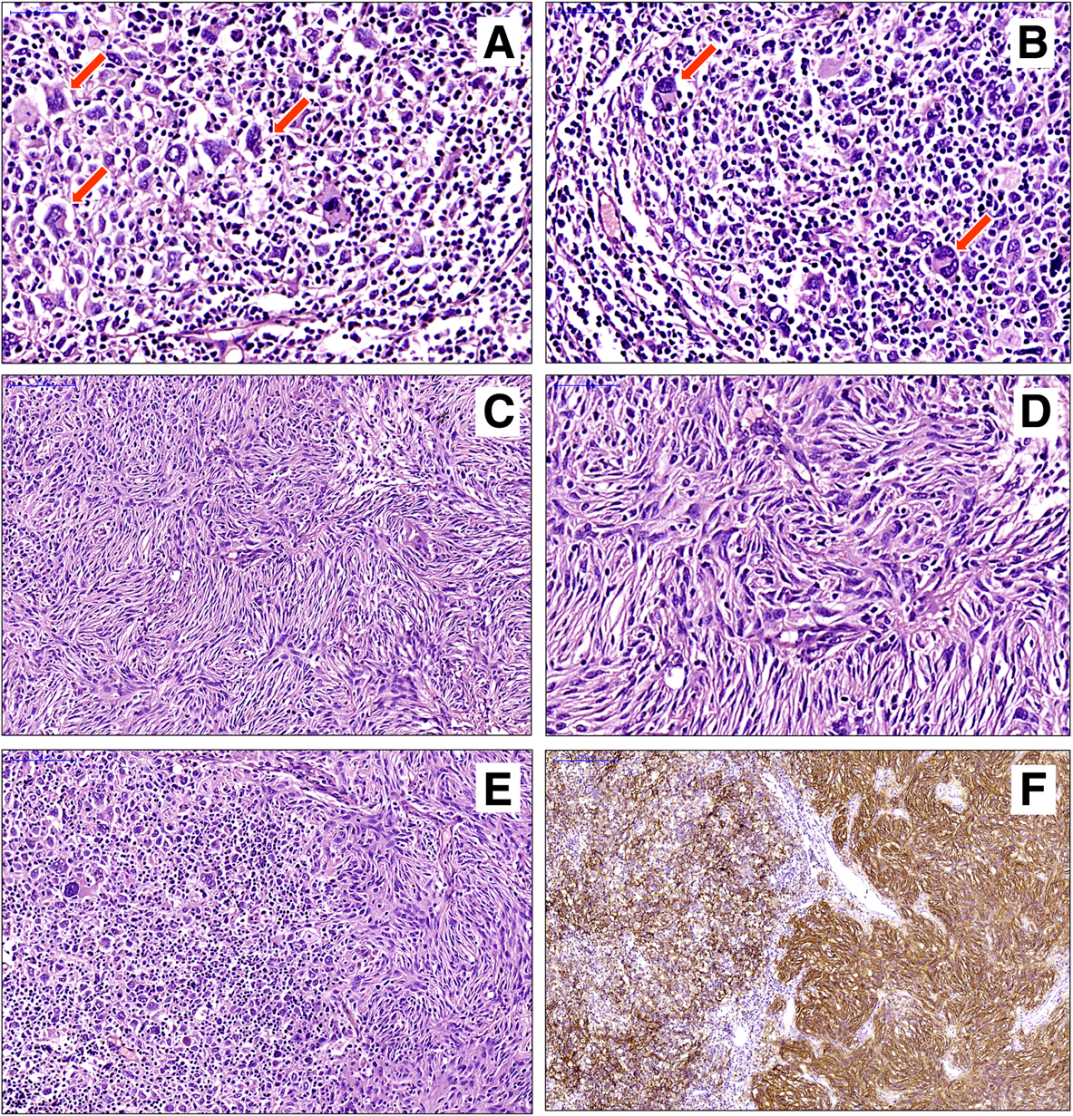
Mononuclear or binuclear Reed-Sternberg (R-S) cell-like large cells (arrows) are scattered in a background composed of lymphocytes and histiocytes, which resembles classical Hodgkin’s lymphoma **a**, **b**. Low- and high-power views show the typical histology of FDCS, which consists of ovoid- to spindle-shaped cells in a storiform pattern (**c**, **d**). In some regions, the transition between the areas, such as those of classical Hodgkin’s lymphoma and those with the typical morphology of FDCS, can be found (**e**), both of which are positive for CD21 (**f**)

On microscopic examination, five distinct histopathologic morphologies could be seen in the resected specimen: (1) Round to polygonal epithelioid tumor cells with marked cellular atypia were arranged in nested or pseudo-glandular structures. Atypical mitoses and multifocal necrosis could also be seen. The epithelioid tumor cell nests infiltrated into hyperplastic collagen fibers, which resembled desmoplastic infiltrative carcinoma. This morphology accounted for approximately 30% of the tumor (Fig. 4a-c). (2) Atypical large tumor cells with a nested or patchy pattern filled the lymphatic sinuses. These cells were round or polygonal with light eosinophilic cytoplasm, granular chromatin and distinct nucleoli. This morphology was similar to that of anaplastic large cell lymphoma, which accounted for about another 30% of the tumor (Fig. 4d-f). (3) Short spindle-shaped tumor cells were arranged radially around many fissure-like or dendritic thin-walled blood vessels with only a few infiltrating lymphocytes. This appearance was essentially indistinguishable from hemangiopericytoma and accounted for approximately 20% of the tumor (Fig. 4g-i). (4) Mononuclear or binuclear Reed-Sternberg (R-S) cell-like large cells were scattered in a background composed of lymphocytes, histiocytes, and a small number of eosinophilic granulocytes; this resembled classical Hodgkin’s lymphoma and accounted for approximately 10% of the tumor (Fig. 5a, b). (5) The typical histology of FDCS was also observed in focal areas, which comprised approximately 10% of the tumor, and consisted of ovoid- to spindle-shaped cells arranged in a storiform or fascicular pattern (Fig. 5c, d). These five distinct morphologies described above were mostly clearly demarcated from each other, but transitions could be seen in some regions (Fig. 5e). IHC showed that tumor cells of all morphologies were positive for CD21 (Fig. 5f), CD23, CD35, CXCL-13 and D2–40 to varying degrees. The Ki-67 labeling index was 20, 30, 10, 20 and 10%, respectively.

**Fig. 6:**
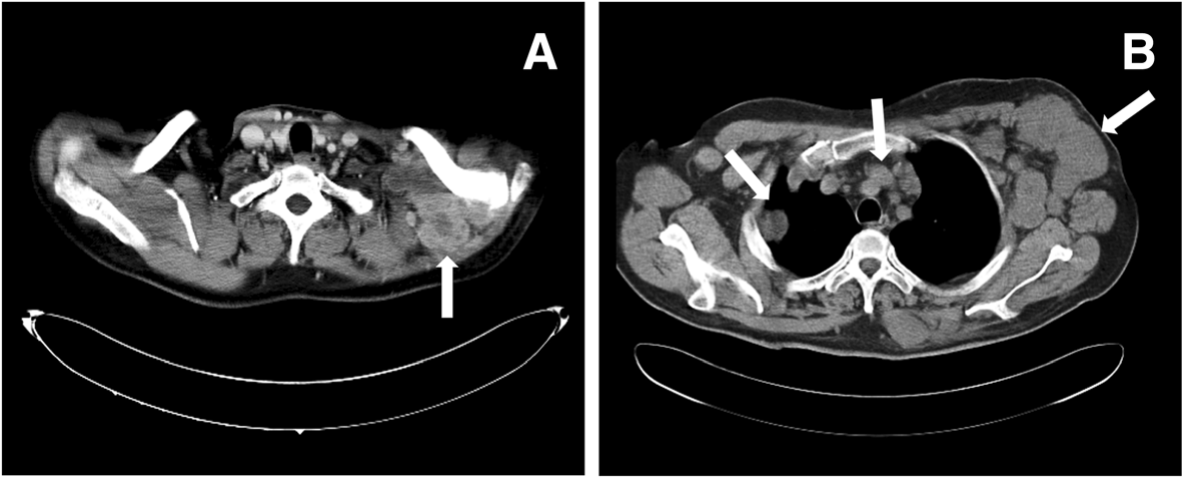
CT shows multiple fused nodular masses in the left supraclavicular fossa with necrosis 17 months after surgery (**a**, arrow). Subsequently, multiple nodules in the bilateral axilla, lungs and mediastinum are found 27 months after operation (**b**, arrows)

After surgery, the patient received two cycles of pharmorubicin and oxaliplatin chemotherapy, which was stopped prematurely due to bone marrow suppression, followed by two months of radiation. After 17 months of follow-up, the patient was found to have a hard nodule at the incision site. CT revealed multiple fused nodular masses in the left supraclavicular fossa with necrosis (Fig. 6a), which suggested tumor recurrence. Despite resumption of radiation therapy, the patient’s condition progressed. At 27 months after surgery, chest CT showed multiple nodules in the bilateral axilla, lungs, and mediastinum (Fig. 6b), which indicated tumor metastasis. The patient refused further treatment and died at 36 months after surgery.

## Discussion

This case posed a variety of diagnostic challenges. One such challenge was the location of the tumor in the left supraclavicular fossa and its atypical histological appearance. In elderly patients, enlarged lymph nodes at this site are most commonly associated with metastasis, particularly of gastric cancer or lung cancer. Moreover, the morphology observed in the biopsy specimen seemed consistent with metastatic carcinoma, which resulted in the initial diagnosis. This was only doubted after no primary lesion was found on further evaluation. Pailoor J et al. reported a case of FDCS in inguinal lymph nodes, which was also initially diagnosed as metastatic carcinoma, even in the surgically resected specimen [4]. The teaching point of this case is that although the epithelioid tumor cell nests had infiltrated into the hypertrophic collagenous stroma, the tumor cells were relatively bland with abundant cytoplasm and lacked crowded or overlapping nuclei. Furthermore, multinucleated giant cells could be readily observed. These morphologic features may be useful in distinguishing FDCS from metastatic carcinoma.

Most notably, this FDCS case demonstrates a spectrum of morphologies. The histomorphology of FDCS is usually heterogeneous and has been known to show epithelioid or pleomorphic tumor cells [5–8], myxoid stroma [9, 10], osteoclastic giant cells [11, 12], and CHL-like features [6, 13], among others. However, in this case, the four atypical histopathologic morphologies were all present within the same tumor, which has not been previously reported. In addition, to the best of our knowledge, the morphological variants resembling ALCL and hemangiopericytoma are described here for the first time.

Discussion of this case will help pathologists to better understand this heterogeneous tumor and to avoid diagnostic error. The first preoperative biopsy may have involved the resection of just the lymph node containing invasive epithelioid cell nests, which resulted in the initial misdiagnosis. The areas similar to ALCL or CHL but that were CD30-positive by IHC (it has been reported that a few FDCS could express CD30 [6, 13]) are another point of diagnostic importance. The treatment of FDCS is typically surgical resection, whereas lymphoma is treated with chemotherapy and radiation. Furthermore, if the IHC results were negative for CD30, CD15, PAX-5, CD20, CD3, EMA and LCA, it would be very difficult to make a definitive diagnosis without knowledge of these FDCS variant morphologies.

No gold standard treatment has been established for FDCS. Radical surgical resection is most often the first treatment choice, and the benefits of adjuvant therapy are controversial. Current studies suggest that patients who undergo surgery alone have a better overall survival than those that receive other treatments and that postoperative adjuvant radiation has no benefit [14]. However, Spatola C et al. [15] demonstrated that adjuvant therapy could strengthen the local control of the disease; Pang J et al. [16] also showed that surgery alone was associated with higher rates of relapse than postoperative adjuvant therapy. Considering the adverse factors [2, 13] in our case including that the maximum tumor diameter was ≥6 cm and that coagulative necrosis, cells with marked atypia, and a focally high mitotic count (> 10/10 high-power fields (HPF)) were observed, the patient received chemotherapy and radiotherapy after radical resection. The patient’s condition was initially well controlled until premature discontinuation of chemotherapy resulted in relapse; subsequent radiation did not effectively control disease progression. At least in this patient’s case, postoperative adjuvant therapy did help control disease burden but not after relapse. At this time, whether patients with recurrent tumors should undergo repeat resection requires further study.

## Conclusion

We report an unusual case of FDCS in the left supraclavicular lymph node with four atypical histomorphologies. These morphologic variants could present a great diagnostic challenge. A better understanding of the variants and familiarity with the immunophenotype will help in the diagnosis of FDCS.

## Data Availability

The datasets used and/or analyzed during this study are available from the corresponding author on reasonable request.

## Abbreviations

ALCL: Anaplastic large cell lymphoma
ALK: Anaplastic lymphoma kinase
CgA: Chromogranin A
CHL: Classical Hodgkin’s lymphoma
CK: Cytokeratin
CT: Computed tomography
EMA: Epithelial membrane antigen
FDC: Follicular dendritic cell
FDCS: Follicular dendritic cell sarcoma
IHC: Immunohistochemistry
LCA: Leukocyte common antigen
MRI: Magnetic resonance imaging
PET-CT: Positron emission tomography and CT
SMA: Smooth muscle actin
Syn: Synaptophysin

## References

[1] Monda L, Warnke R, Rosai J (1986). A primary lymph node malignancy with features suggestive of dendritic reticulum cell differentiation. A report of 4 cases. Am J Pathol.

[2] Chan JK, Fletcher CD, Nayler SJ, Cooper K (1997). Follicular dendritic cell sarcoma: Clinicopathologic analysis of 17 cases suggesting a malignant potential higher than currently recognized. Cancer..

[3] Duan GJ, Wu F, Zhu J (2010). Extranodal follicular dendritic cell sarcoma of the pharyngeal region: a potential diagnostic pitfall, with literature review. Am J Clin Pathol.

[4] Pailoor J, Iyengar KR, Chan KS, Sumithra S (2008). Follicular dendritic cell sarcoma of inguinal lymph node-a case report. Malays J Pathol.

[5] Viola P, Vroobel KM, Devaraj A (2016). Follicular dendritic cell tumour/sarcoma: a commonly misdiagnosed tumour in the thorax. Histopathology..

[6] Facchetti F, Pileri SA, Lorenzi L (2017). Histiocytic and dendritic cell neoplasms: what have we learnt by studying 67 cases. Virchows Arch.

[7] Duan GJ, Wu YL, Sun H, Lang L, Chen ZW, Yan XC (2017). Primary follicular dendritic cell sarcoma of the urinary bladder: the first case report and potential diagnostic pitfalls. Diagn Pathol.

[8] Wu B, Lim CM, Petersson F. Primary tonsillar epithelioid follicular dendritic cell sarcoma: report of a rare case mimicking undifferentiated carcinoma and a brief review of the literature. Head Neck Pathol. 2019. 10.1007/s12105-019-01015-3 Epub ahead of print. 10.1007/s12105-019-01015-3 PMC6854199 30758753

[9] Fisher C, Magnusson B, Hardarson S, Smith ME (1999). Myxoid variant of follicular dendritic cell sarcoma arising in the breast. Ann Diagn Pathol.

[10] Pruneri G, Masullo M, Renne G (2002). Follicular dendritic cell sarcoma of the breast. Virchows Arch.

[11] Shen SC, Wu CC, Ng KF, Wu RC, Chen HM, Chen TC (2006). Follicular dendritic cell sarcoma mimicking giant cell carcinoma of the pancreas. Pathol Int.

[12] Vaideeswar P, George SM, Kane SV, Chaturvedi RA, Pandit SP (2009). Extranodal follicular dendritic cell sarcoma of the tonsil-case report of an epithelioid cell variant with osteoclastic giant cells. Pathol Res Pract.

[13] Wu YL, Wu F, Xu CP (2019). Mediastinal follicular dendritic cell sarcoma: a rare, potentially under-recognized, and often misdiagnosed disease. Diagn Pathol.

[14] Saygin C, Uzunaslan D, Ozguroglu M, Senocak M, Tuzuner N (2013). Dendritic cell sarcoma: a pooled analysis including 462 cases with presentation of our case series. Crit Rev Oncol Hematol.

[15] Spatola C, Migliore M, Emanuele Liardo RL (2015). Follicular dendritic cell sarcoma of mediastinum: a key role of radiotherapy in a multidisciplinary approach. Future Oncol.

[16] Pang J, Mydlarz WK, Gooi Z (2016). Follicular dendritic cell sarcoma of the head and neck: case report, literature review, and pooled analysis of 97 cases. Head Neck.

